# Controlled infection with cryopreserved human hookworm induces CTLA-4 expression on Tregs and upregulates tryptophan metabolism

**DOI:** 10.1080/19490976.2024.2416517

**Published:** 2024-10-16

**Authors:** Francesco Vacca, Thomas C. Mules, Mali Camberis, Brittany Lavender, Sophia-Louise Noble, Alissa Cait, Kate Maclean, John Mamum, Bibek Yumnam, Tama Te Kawa, Laura Ferrer-Font, Jeffry S. Tang, Olivier Gasser, Graham Le Gros, Stephen Inns

**Affiliations:** a Le Gros Laboratory, Malaghan Institute of Medical Research, Wellington, New Zealand; b Department of Medicine, University of Otago, Wellington, New Zealand; c Gastroenterology, Te Whatu Ora, Capital Coast and Hutt Valley, Wellington, New Zealand; d Hugh Green Technology Centre, Malaghan Institute of Medical Research, Wellington, New Zealand

**Keywords:** Hookworm, helminths, microbiome, immune regulation, eosinophilia, clinical trial, cryopreservation, controlled infection

## Abstract

Infecting humans with controlled doses of helminths, such as human hookworm (termed hookworm therapy), is proposed to prevent or treat various intestinal and extraintestinal diseases. However, full-scale clinical trials examining hookworm therapy are limited by the inability to scale-up the production of hookworm larvae to infect sufficient numbers of patients. With the aim of overcoming this challenge, this study infected four healthy individuals with hookworm larvae that had been reanimated from cryopreserved eggs to examine their viability and immunogenicity. We demonstrate that reanimated cryopreserved hookworm larvae establish a viable hookworm infection and elicit a similar immune response to larvae cultured from fresh stool. Furthermore, a refined understanding of the therapeutic mechanisms of hookworm is imperative to determine which diseases to target with hookworm therapy. To investigate potential therapeutic mechanisms, this study assessed changes in the immune cells, microbiome, and plasma metabolome in the four healthy individuals infected with cryopreserved hookworm larvae and another nine individuals infected with larvae cultured from freshly obtained stool. We identified potential immunoregulatory mechanisms by which hookworm may provide a beneficial effect on its host, including increased expression of CTLA-4 on regulatory T cells (Tregs) and upregulation of tryptophan metabolism. Furthermore, we found that a participant’s baseline microbiome predicted the severity of symptoms and intestinal inflammation experienced during a controlled hookworm infection. In summary, our findings demonstrate the feasibility of full-scale clinical trials examining hookworm therapy by minimizing the reliance on human donors and optimizing the culturing process, thereby enabling viable hookworm larvae to be mass-produced and enabling on-demand inoculation of patients. Furthermore, this study provides insights into the complex interactions between helminths and their host, which could inform the development of novel therapeutic strategies.

## Introduction

Intestinal helminths are multicellular organisms that have co-evolved with their human hosts over millennia. The observation that infection with some species of helminths can last for years and are generally well tolerated suggests a state of host immunological tolerance.^1^ Over the past 20 years, researchers’ interest in harnessing the associated immune changes to treat a range of diseases has grown. An example of this mutually beneficial host-helminth interaction is the human hookworm, *Necator americanus*. Human clinical trials using controlled doses of hookworm, an approach termed controlled hookworm infection, have been conducted in ulcerative colitis, Crohn’s disease, multiple sclerosis, metabolic disease, celiac disease, and asthma, with some therapeutic success.^2–8^

The use of hookworm as a therapeutic agent offers multiple potential benefits. It could protect the host from various diseases, including allergic, inflammatory, autoimmune, and metabolic conditions. Many regard it as a ‘natural’ alternative to conventional therapies. Additionally, helminths, when administered in controlled doses, are safe and usually well tolerated. Significantly, *N. americanus* has the capacity to remain in the body for several years. This means that a single dose might provide long-lasting benefits, potentially eliminating the need for frequent treatments.^1,7^ These significant clinical advantages have been the driving force behind continued efforts by clinicians and researchers to investigate hookworm therapy, including establishing its mechanism of action. Although a well-defined mechanism through which helminths protect their host from disease is yet to be identified and characterized, prominent hypotheses include an increase in the production of regulatory immune cells and cytokines and favorable changes in the microbiome.^1^

One complexity of examining hookworm therapy in full-scale clinical trials is scaling up the production of hookworm larvae to infect large numbers of patients. Current methods to infect an individual with hookworm require fresh stool from a human donor.^9^ The use of reanimated cryopreserved hookworm larvae is a plausible method to reduce the reliance on human donors and speed up the culturing process, meaning viable hookworm larvae could be mass-produced and patients could be inoculated on-demand. To examine the viability and immunogenicity of hookworm reanimated from cryopreserved larvae, and investigate potential therapeutic mechanisms through which hookworms elicit their benefit on human health, this study infected 13 healthy individuals with 30 *N. americanus* larvae either cultured from freshly obtained stool (‘fresh larvae’) or reanimated from stores of cryopreserved larvae (‘cryopreserved larvae’), and assessed infection viability and changes in the immune networks, microbiome, and plasma metabolome. This study’s findings make performing full-scale clinical trials examining hookworm therapy more feasible by reducing the reliance on human donors and optimizing the culturing process, meaning viable hookworm larvae can be mass-produced and patients can be inoculated on-demand. Furthermore, this study provides insights into the complex interactions between helminths and their host, which could inform the development of novel therapeutic strategies for a range of autoimmune and inflammatory conditions linked to the gut ecosystem.

## Results

### A viable and durable controlled hookworm infection is established from reanimated cryopreserved larvae

**Figure 1.**
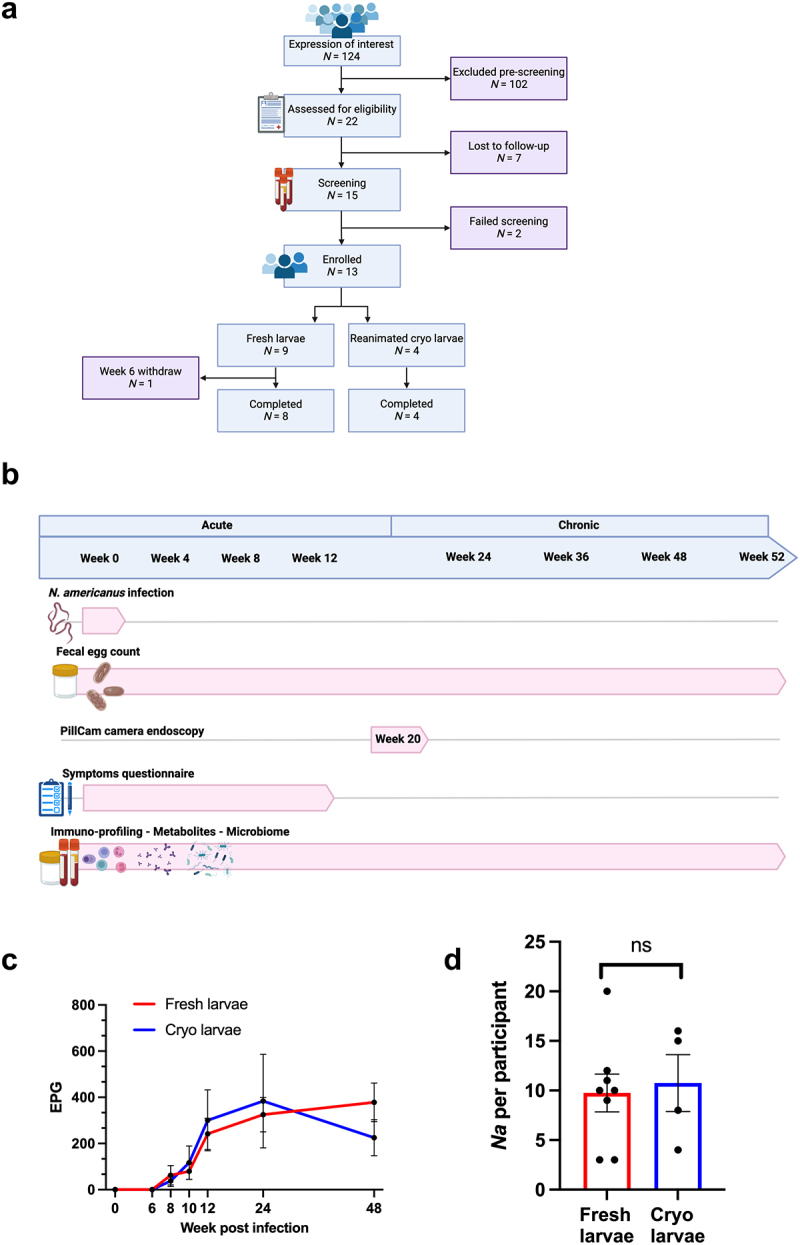
A viable and durable controlled hookworm infection is established from reanimated cryopreserved larvae. (a) Schematic representation of the recruitment and eligibility process. Eligibility criteria described in data file S1. (b) Study design with sample collection. (c) Hookworm eggs count (eggs/gram) measured in fresh stool samples in participants that received fresh (red line) or cryopreserved (blue line) larvae. Mean ± SEM. (d) Adult hookworm counted in the gastrointestinal tract with PillCam^TM^ endoscopy in participants that received fresh (red) and cryopreserved (blue) larvae. Data were analyzed with the unpaired Student’s t-test.

Thirteen healthy individuals (7 female; median age 47 years-old, range 19–64) were inoculated with 30 *N. americanus* larvae and followed-up for 12 months. Nine participants received fresh larvae and four participants received reanimated cryopreserved larvae. One participant who received fresh larvae withdrew from the study at week 6 post-infection and was excluded from subsequent analyses (Figures 1(a,b)). A patent hookworm infection was detected in every participant as evidenced by detectable eggs in fecal samples from week 8 post-infection onwards (Figure 1(c)), mature hookworm visualized on PillCam^TM^ capsule endoscopy at week 20 (mean number of visualized worms = 10 worms, range 3–20) (Figure 1(e) and S1A), and the detection of *Necator americanus* specific IgG at week 24 post-infection (Figure S1B). No significant differences in infection burdens were observed between individuals infected with fresh and cryopreserved larvae (Figures 1(c,d)).

All participants had detectable eggs in their fecal samples at week 48 post-infection indicating a durable infection (Figure 1(c)). Six individuals, three who received fresh larvae and three who received cryopreserved larvae, entered the continuation phase of the study. The fecal eggs counts remained positive in all these individuals until they exited the study (mean follow-up period 27 months post-infection, range 19–35, and mean egg count on exiting the study 250 eggs/g, range 50–550).

Self-reported symptoms during the infection were consistent with the lifecycle of *N. americanus* which includes transit through the skin and lungs to reach the gut. During the first few weeks, all participants experienced skin reactions (e.g., rash and itchiness) and one participant experienced minor respiratory symptoms (e.g., cough, sneezing or sore throat). Most participants experienced transient gastrointestinal symptoms at 5- and 6-weeks post-infection (Figure S1C). One patient withdrew at week-6 post-infection due to intolerability of moderate gastrointestinal symptoms (Figure 1(a)). All symptoms resolved in the chronic infection phase and no severe reactions were reported, indicating good tolerability of a chronic hookworm infection.

### Hookworm infection induces a systemic and intestinal type 2 immune response

**Figure 2.**
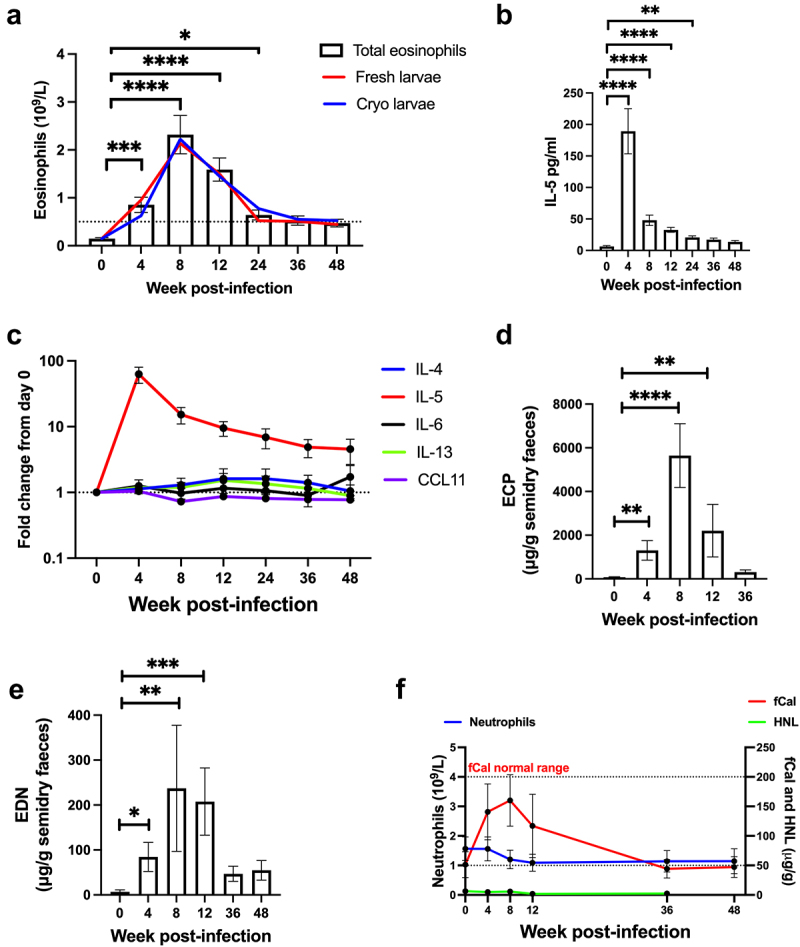
Hookworm infection induces a systemic and intestinal type 2 immune response (b) mean (±SEM) peripheral blood eosinophil count in participants infected with fresh (red line) or cryopreserved (blue line) larvae. (b) Mean (±SEM) serum IL-5 levels in all participants. (c) Fold change in type 2 cytokines, IL-6 and eotaxin-1 (CCL11) from baseline (day 0= pre-infection) in all participants. (d) Mean (±SEM) faecal eosinophilic cationic protein (ECP) in all participants. (e) mean (±SEM) faecal eosinophil derived neurotoxin (EDN) in all participants. f) mean (±SEM) faecal calprotectin (fCal – red line), neutrophils count (blue line) and human neutrophil lipocalin (HNL) (green line). Data were analysed with one-way ANOVA using Friedman test with Dunn’s multiple comparison test. *p*= * < 0.05, ** < 0.005, *** < 0.001, **** < 0.0001.

All participants experienced an increase in blood eosinophils (peak at week 8) and serum IL-5 (peak at week 4) throughout the follow-up period (Figures 2(a,b)), indicating a systemic Type 2 immune response. Of note, no significant changes were observed in the cytokines IL-13 and IL-4, or the chemokine eotaxin/CCL11 (Figure 2(c)). No significant difference in blood eosinophils was observed between participants infected with fresh larvae or cryopreserved larvae (Figure 2(a)). Given that no differences were observed in eggs counts, visualized worms and eosinophil kinetics using fresh or reanimated cryopreserved larvae, all further analyses were carried out as a combined set.

To assess if an intestinal Type 2 immune response is also induced by hookworm, fecal levels of the eosinophilic activation and degranulation proteins, eosinophilic cationic protein (ECP) and eosinophil-derived neurotoxin (EDN), were measured. An increase in both ECP and EDN was observed from week 4 post-infection, with levels peaking at week 8 (Figures 2(d,e)). A significant increase in fecal calprotectin (fCal) was also observed in the acute infection phase, however no increase in fecal human neutrophil lipocalin (HNL), a specific neutrophil degranulation marker, was observed. Similarly, no significant increase in blood neutrophils was observed (Figure 2(f)). These findings indicate the source of fCal is from eosinophils rather than neutrophils. These data indicate that a controlled hookworm infection induces a systemic and intestinal Type 2 immune response and support eosinophils as the predominant innate immune cell activated in the intestine during hookworm infection.

Interestingly, participants that experienced greater gastrointestinal symptoms also demonstrated a greater increase in fCal, as well as a trend toward a greater increase in fecal ECP and EDN, compared to patients that experienced no or mild gastrointestinal symptoms (Figure S2A, S2B, and S2C). These data suggest that eosinophilic inflammation arising as part of the intestinal Type 2 inflammatory response against hookworm may contribute to gastrointestinal symptoms experienced by individuals infected with controlled hookworm infections.

There were no significant changes in cytokines involved in type 1 immune responses (Figure S2D), chemokines (Figure S2E), serum antibodies (Figure S2F), or the immunoregulatory cytokines, TGF-β1(Figure S2G) and IL-10 (Figure S2H), during the controlled hookworm infection.

### Gastrointestinal symptomatology may depend on the baseline microbiome

**Figure 3.**
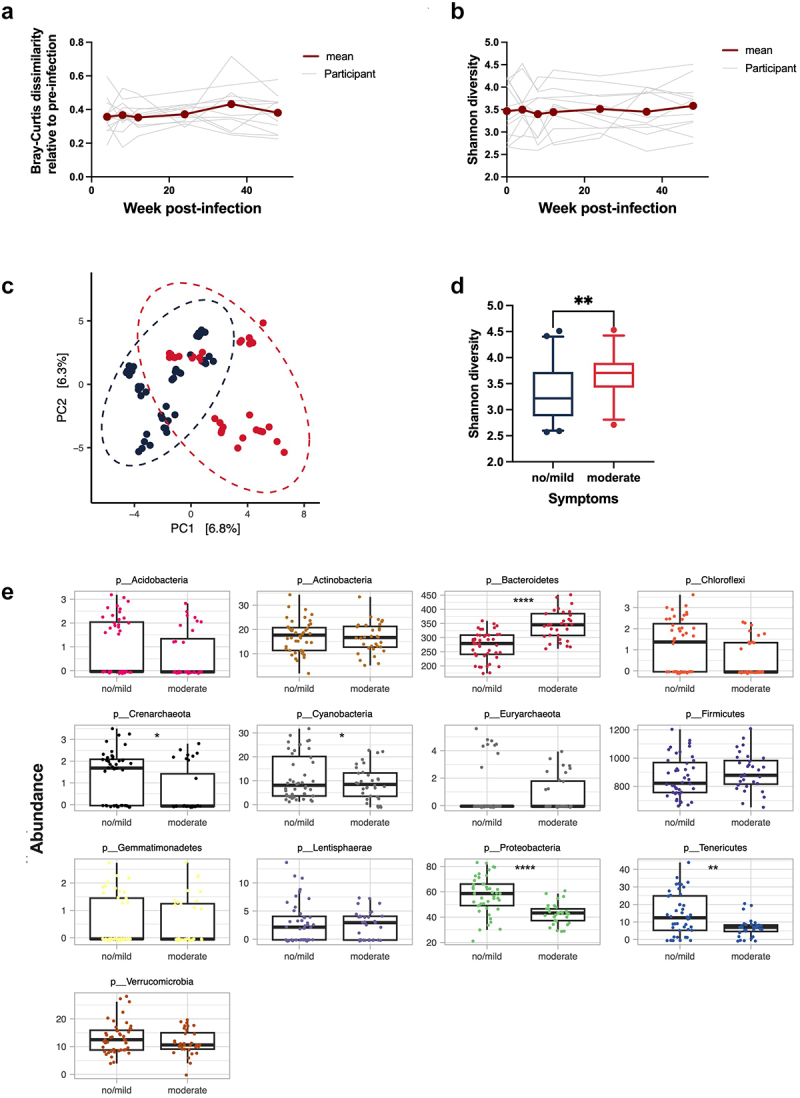
Gastrointestinal symptomatology may depend on the baseline microbiome (a) changes in Bray-Curtis dissimilarity test for each single participant (gray lines) and mean (red line). (b) Changes in Shannon diversity for each participant (gray lines) and mean (red line). (c) PCA plot showing 16s analysis grouping participants according to symptoms score (no/mild symptoms (blue); moderate symptoms (red)). (d) Box plot showing Shannon diversity according to the severity of symptoms (no/mild symptoms (dark blue) and moderate symptoms (red)). (e) Comparison in the relative abundance of the top 100 most abundant taxa faceted by phylum in participants who experienced no or mild symptoms (symptom score 0) and participants who experience moderate symptoms (symptom score 1).

The fecal microbiome was analyzed during the acute (week 8 post-infection) and chronic (week 36 post-infection) infection phases. No significant changes to beta-diversity were induced by the hookworm infection (PERMANOVA, *p* > 0.1). Similarly, microbial community stability (Bray-Curtis dissimilarity relative to baseline) (Figure 3(a)) and alpha diversity (Shannon diversity index) (Figure 3(b)) were unchanged post-infection. When beta-diversity was visualized by principal component analysis (PCA), the inter-individual microbiome composition was demonstrated to be the greatest source of variation in the data (Fig S3A).

Previous studies have demonstrated an association between changes in the microbiome and the severity of gastrointestinal symptoms experienced by the participants, so this was also examined in the current study.^10^ Interestingly, participants that experienced moderate/severe gastrointestinal symptomology (Figure 3(c), shown in red) had a significantly different baseline microbiome from those who experienced mild or no symptoms (shown in dark blue; PERMANOVA *p* < 0.001) (Figure 3(c)). Participants with moderate/severe symptoms demonstrated higher alpha-diversity, greater abundance of the phylum Bacteroides, and at an OTU level, 43 differentially abundant taxa (Figures 3(d,e), S3B, and S3C, Table S1 and S2). Furthermore, a trend toward a positive correlation between baseline alpha diversity and fecal calprotectin (fCal) and eosinophilic cationic protein (ECP) levels was observed at week 8 post-infection, suggesting a higher baseline alpha diversity may be associated with increased intestinal inflammation during a hookworm infection (Figure S3D).

### Hookworm infection upregulates the immune checkpoint receptor CTLA-4 on tregs

**Figure 4.**
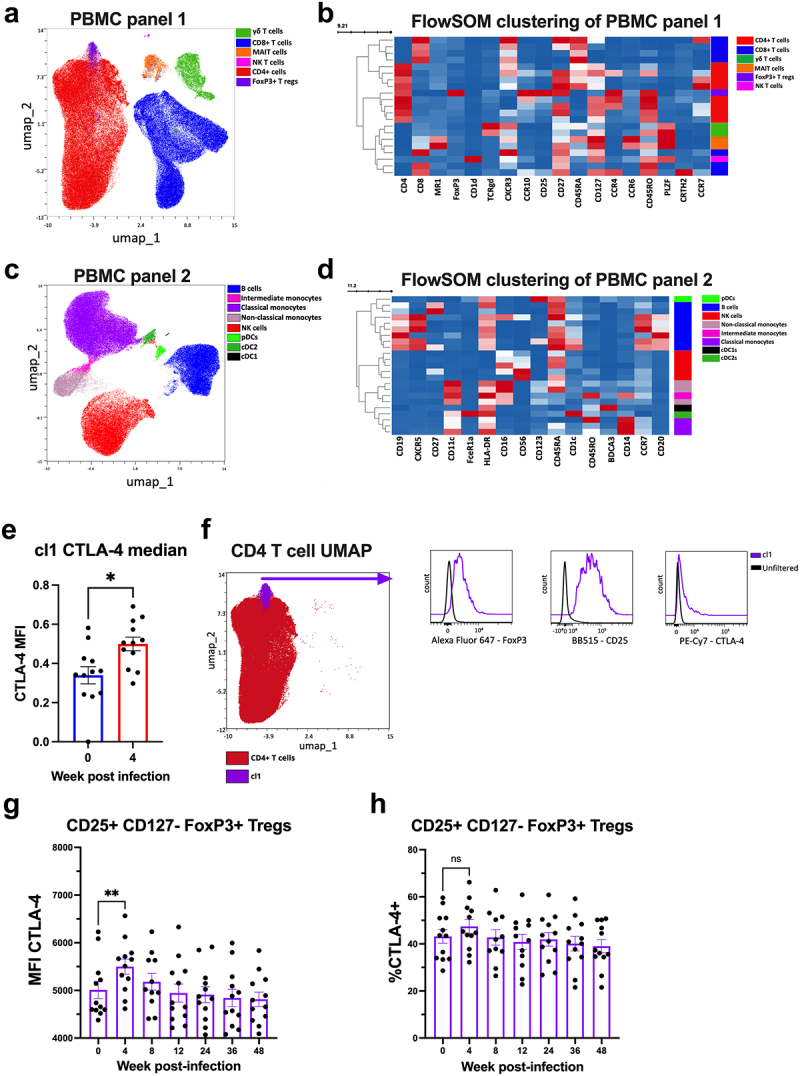
Hookworm infection upregulates the immune checkpoint receptor CTLA-4 on tregs. PBMC samples were collected at baseline and weeks 4, 8, 12, 36, and 48. Two flow cytometry panels were used to analyze immune cells using OMIQ software to generate: (a) UMAPs showing the main PBMC populations identified using panel 1 overlaying all timepoints; (b) all timepoints from panel 1 were analysed using FlowSOM. Heatmap showing median FlowSOM cluster marker expression for panel 1 (red=high expression, blue= low expression). According to the marker expression, clusters were assigned a cell population as identified with the colored column on the right side of the heatmap; (c) UMAPs showing the main populations identified using panel 2 overlaying all timepoints; (d) all timepoints from panel 2 were analysed using FlowSOM. Heatmap showing the markers expression of each cluster identified with FlowSOM (red=high expression, blue= low expression). According to the marker expression, clusters were assigned a cell population as identified with the colored column on the right side of the heatmap. Clusters generated by FlowSOM in (b) were analysed for markers expression showing (e) cluster 1 median CTLA-4 expression comparison between week 0 (pre-infection) and week 4 post-infection. (f) UMAP showing the main CD4 T cell population overlapping FlowSOM cluster 1. Histograms showing FoxP3, CD25 and CTLA-4 expression for cluster 1. (g) CTLA-4 MFI of T_REG_ using expert gating during infection. (h) frequency of CTLA-4^+^ T_REG_ using expert gating. Analyzed using the paired t-test (e) or one-way ANOVA with mixed-effects analysis with Tukey’s multiple comparison test (g and h). *p*= *<0.05, **<0.005.

Peripheral blood mononuclear cells (PBMCs) were analyzed by high-dimensional flow cytometry using an unbiased High-Dimensional Analysis approach, as well as using expert gating strategies (Figure S4). Normalization was performed to remove batch to batch variation. All timepoints were included during the analysis using FlowSOM and Uniform Manifold Approximation and Projection (UMAP) to reduce the dimensions of the data and to map immune cells (Figure 4(a-d)).

Two panels were used to analyze PBMC samples. To visualize the main populations for panel 1 and for panel 2, all PBMC timepoints were plotted using UMAP (Figures 4(a,c)) and, according to their receptor expression, we identified CD4^+^ and CD8^+^ T cells, γδT cells, T_REG_, MAIT cells, and NKT cells for panel 1 (Figure 4(a)), and B cells, dendritic cells, NK cells, and monocytes for panel 2 (Figure 4(c)). UMAPs generated were also used to display main receptor expression (Figure S5A and S5H). Then, a heatmap was created to visualize the marker expression of each cluster generated by FlowSOM for both panel 1 (Figure 4(b)) and panel 2 (Figure 4(d)). Using expert gating (Figure S4), no significant changes were observed in all these main cell populations (Figure S5B and Figure S5I). Similarly, no significant differences were detected when FlowSOM cluster frequencies were assessed using Significance Analysis of Microarrays (SAM) for each time point (Figure S5C and Figure S5K).

While FlowSOM clustering analysis was performed using all timepoints, we used SAM to investigate differences in receptor expression for each FlowSOM cluster during the infection. For panel 1, SAM identified a significant increase in the expression of cytotoxic T lymphocyte antigen 4 (CTLA-4) in cluster 1 at week 4 post-infection (Figure 4(e)). There were no significant changes in CTLA-4 in cluster 1 at the other timepoints. As demonstrated in the UMAP (Figure 4(f)), cluster 1 is associated with CD4^+^ T cells and express FoxP3 and CD25, identifying the cells in cluster 1 as T_REG._ Using expert gating, the CD25^+^ CD127^−^ FoxP3^+^ population had increased expression of CTLA-4 at this timepoint (Figure 4(g)), however no increase in the frequency of CTLA-4+ T_REG_ was observed (Figure 4(h)). Notably, CTLA-4 expression was also increased in CD4^+^ CCR4^+^ CXCR3^−^ T_H_2 (Figure S5J) and CD4^+^ CCCR6^+^ CCR4^−^ CXCR3^−^ T_H_9 (Figure S5L) at 8-weeks and 12-weeks post-infection, respectively. Increased expression of CTLA-4 was not associated with an increase in CTLA-4+ T_H_2 or T_H_9 frequency (Figure S5M and S5N). No significant changes in marker expression were observed in the other clusters.

### Hookworm infection upregulates tryptophan metabolism

**Figure 5.**
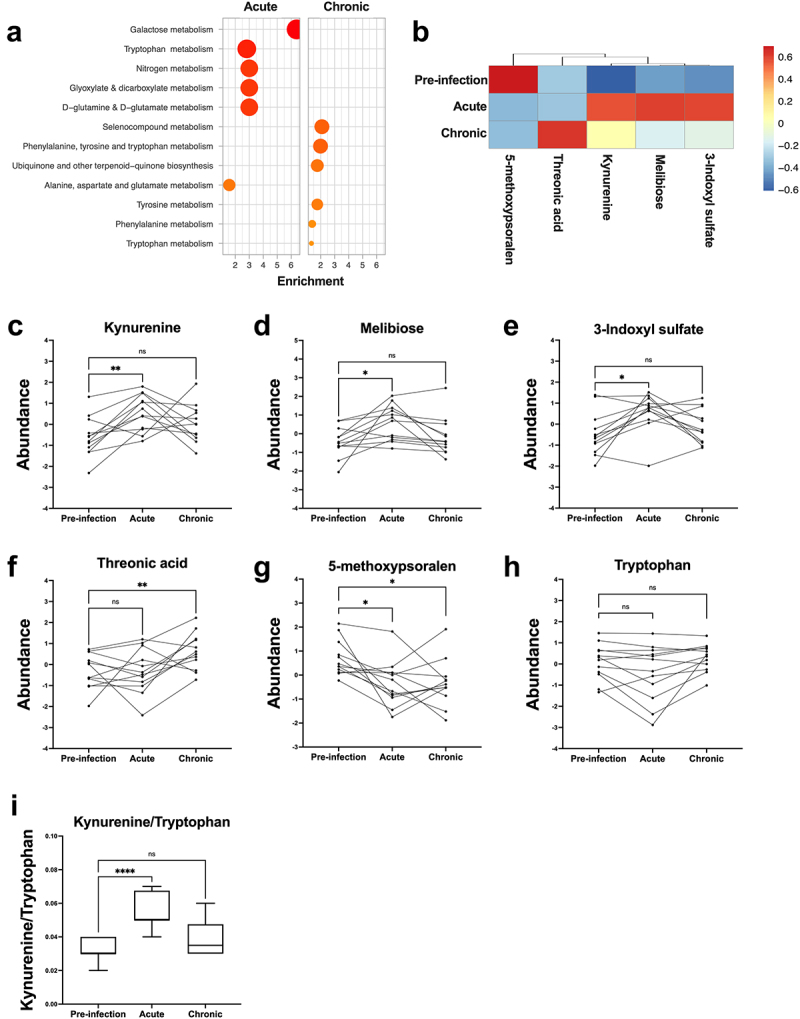
Hookworm infection upregulates tryptophan metabolism. (a) Top six enriched metabolic pathways identified using MSEA comparing pre-infection vs acute phase (left panel) or chronic phase of infection (right panel). (b) Heatmap of five annotated plasma metabolites which significantly (*p* < 0.05) changed during the hookworm infection, as determined by mixed-mode, two-way analysis of variance (ANOVA), controlling for ‘participant ID’ and ‘sex’ as covariates.(c) changes in plasma kynurenine (c), melibiose (d), indoxyl sulphate (e), threonic acid (f), 5-methoxypsoralen (f), and tryptophan (h) levels during the hookworm infection. (i) changes in plasma kynurenine/tryptophan ratio, a marker of indoleamine 2,3-dioxygenase (IDO) activity. Analysed with mixed ANOVA with Dunnett’s. Values shown represent log_10_ transformed (*N* = 12 participants). *p* = *< 0.05, **< 0.005, ****< 0.0001.

Untargeted metabolomics with Metabolic Set Enrichment Analysis (MSEA) was performed on plasma samples to analyze changes during the acute and chronic phases of the hookworm infection. Based on mass spectral database matching with MS-DIAL’s built-in metabolomics databases (Data File S1), a total of 12 annotated metabolites including heme, amino acids, and amino acid metabolic by-products were detected (Figure 5(a)). A total of 94 unique metabolites were annotated, broadly encompassing a diversity of chemical classes including amino acids, nucleotides, amino acid metabolism products, organic acids, some dietary-related alkaloids, and exogenous drugs (Data File S1). Investigation of the sample variation after correction for run-order using QC samples highlighted excellent quality of the data (Figure S6B).

The acute infection phase was associated with a significant increase in the enrichment of metabolites relevant to galactose and tryptophan metabolic pathways (Figure 5(a); Table S3). In addition, metabolic pathways involving amino acids (glutamine and glutamate; alanine and aspartate), glyoxylate, and dicarboxylate compounds were also enriched, although these changes were not significant (Figure 5(a); Data File S1). In the chronic infection phase, non-significant increases in selenocompound metabolism, biosynthetic pathway of amino acids, metabolism of amino acids, and biosynthesis of ubiquinone and/or terpenoid-quinones (Figure 5(a); Table S4) were observed.

To interpret these data in a biologically relevant manner, complementary statistical methods that control for potential confounding covariates were performed. PERMANOVA analysis revealed that the participant gender was the greatest source of variation in the data (PERMANOVA, *p* < 0.001) (Figure S6C, Table S5), followed by inter-individual human variation (PERMANOVA, *p* = 0.072) (Figure S6C, Table S5). After controlling for these variables (Table S6), the plasma levels of kynurenine, xenobiotic, melibiose, threonic acid, indoxyl sulfate, and 5-methoxypsoralen were significantly different between at least two of the analyzed time-points (Figure 5(b-h)). Kynurenine, melibiose, and indoxyl sulfate were the major contributors to the top two metabolic pathways (tryptophan and galactose) (Figure 5(a)) and significantly increased in the acute infection phase before returning to levels similar to baseline in the chronic infection phase (Figure 5(b-e)). A trend toward reduced plasma tryptophan levels during the acute phase was also observed (Figure 5(h)). Overall, an increase in the kynurenine/tryptophan ratio was observed (Figure 5(i)). This indicates increased activity of indoleamine 2,3-dioxygenase (IDO), the rate-limiting enzyme responsible for converting tryptophan to kynurenine and for shunting tryptophan down the kynurenine pathway. A decrease in 5-methoxypsoralen in the acute and chronic infection phases (Figure 5(g)) and an increase in threonic acid levels in the chronic phase of infection (Figure 5(f)) were also observed.

## Discussion

The rationale for using helminths, such as hookworm, as a therapy initially emerged from epidemiological data demonstrating an inverse relationship between the incidence of helminth infections and various autoimmune, inflammatory, and allergic diseases. This forms part of the ‘hygiene hypothesis’ and more specifically ‘the old friends’ hypothesis’, which are plausible explanations for the increasing incidence of these diseases world-wide.^1,7^ Several small clinical trials examining controlled hookworm infection to treat disease have now been conducted, with some showing benefit.^5,8^

A barrier to performing full-scale clinical trials is scaling up the production of hookworm larvae to infect large numbers of patients given the need to extract eggs from freshly donated stool. Previous methods to infect an individual with hookworm required fresh stool from a human donor, intensive labor to isolate and culture eggs to obtain viable larvae, and infecting the individual within one to two weeks of hatching before larvae viability starts to decline.^9^ This complex and time-consuming process requires significant planning and expertise, limiting the ability to scale up the production of hookworm larvae. Our study has demonstrated that a viable hookworm infection can be achieved from reanimated cryopreserved hookworm larvae, and that these hookworms elicit a similar immune response to hookworm larvae cultured from eggs obtained from fresh stool. These findings allow banks of cryopreserved hookworm larvae to be utilized to reduce the reliance on human donors and speed up the culturing process, meaning viable hookworm larvae can be mass-produced and patients can be inoculated on-demand.

Previous clinical trials investigating hookworm therapy have failed to successfully establish a viable infection in a sizable proportion of patients inoculated with hookworm.^3,6^ The primary outcome of this study was developing the methodologies to establish a viable infection in all participants. All volunteers had a positive fecal egg counts at week 8 indicating a viable infection. Given microscopic examination of feces for hookworm eggs is only semiquantitative, this study also used capsule endoscopy to quantify intestinal worm burden through direct visualization.^11^ Of the 30 hookworm larvae given to each participant, on average one-third of these established a chronic infection in the intestine. Furthermore, significant variation in intestinal worm burden between participants was observed. Plausible explanations for this include differences in the viability of hookworm larvae given and heterogeneity in the effectiveness of the host’s immune response aimed at helminth expulsion. These findings highlight the challenge of dosing with controlled hookworm therapy to achieve the optimal level of infection.

While preclinical models have provided insight into potential mechanisms of action of helminth therapy, the immunological mechanisms that confer benefit in humans remain unclear. Helminths are reported to regulate inflammation through the induction of an immunoregulatory phenotype within immune cells, particularly within the T cell compartment.^12^ Our study demonstrates that a controlled hookworm infection upregulates the expression of CTLA-4, a checkpoint receptor involved in immunomodulation, on Treg, T_H_2 and T_H_9 cells. This finding is consistent with previous studies examining the immune effects of hookworm in infected individuals living in endemic areas.^13,14^ Importantly, CTLA-4+ Tregs can downregulate Th1 and Th17 mediated inflammation indicating this as a possible mechanism through which hookworm could prevent or treat inflammatory diseases.^15^ In the current study, an increase in CTLA-4 expression was observed only in the acute infection phase before returning to baseline levels in the chronic infection phase. These findings suggest that repeated infections could be required to sustain this immunoregulatory mechanism, like what is experienced by individuals living in endemic countries.

The impact of controlled hookworm infections on the microbiome has been examined in previous studies.^10,16–18^ Results are often conflicting, potentially due to heterogeneity in baseline microbiomes, differences between the dose of hookworm given, and variations in the site and method of sampling. In the current study, no significant changes in the fecal microbiome were observed during a controlled hookworm infection. Interestingly, participants that experienced moderate gastrointestinal symptoms and higher fCal levels had a more diverse microbiome at baseline compared to participants experiencing mild or no symptoms. Given that symptoms in a hookworm infection are predominately caused by the immune response against the invading helminth, these findings may suggest that an individual’s baseline microbiome determines the magnitude of this immune response. These findings should be confirmed with a larger sample size and include microbial metagenomics to better understand functional shifts in the microbiome.

To explore changes in metabolism during a controlled hookworm infection, untargeted metabolomics was performed on plasma samples. An increase in the kynurenine/tryptophan ratio was observed indicating upregulated activity of the rate-limiting enzyme responsible for converting tryptophan to kynurenine, indoleamine 2,3-dioxygenase (IDO). Kynurenines exert important immunosuppressive functions including inducing Treg proliferation and reducing the activity NK and dendritic cells.^19^ A possible mechanism for increased IDO activity during a hookworm infection is the upregulation in CTLA-4 expression on Tregs, which has been demonstrated to increase IDO activity in other immune cells.^15^

In addition to tryptophan metabolism, metabolomic analysis demonstrated an increase in plasma melibiose levels indicating the enrichment of galactose metabolism. Melibiose, a disaccharide of glucose and galactose, suppresses T_H_2 cells and induces oral tolerance in a model of OVA sensitization suggesting it could be involved in regulating the Type 2 inflammatory response during the hookworm infection.^20^ These findings highlight the need for future studies to conduct more in-depth and targeted metabolomic analyses, particularly to determine the role of products of tryptophan metabolism in the immunomodulatory properties of a controlled hookworm infection.

This study highlights hookworms’ ability to reside in the body for several years which means a single dose could potentially deliver a prolonged benefit without the need for repeat dosing. This characteristic of hookworm therapy could help overcome barriers to adherence with daily medications, including: forgetting to take daily medication; not having medicine on-hand due to the cost of visiting a doctor, prescription charges, or lack of access to health care; and pill-burden, particularly when taking multiple medications.^21,22^ For this characteristic of hookworm therapy to be advantageous, its beneficial immunological effects need to persist into the helminths’ chronic infection phase. While the potentially beneficial immune responses identified in the current study (upregulation of CTLA-4 and tryptophan metabolism) appeared to only occur in the acute infection phase, previous research has identified favorable immune responses that persist into the chronic infection phase, raising the potential for hookworm therapy to deliver a prolonged benefit from a single dose.^23^

A limitation of this study was only examining the host’s immune response in the peripheral circulation, which is not always reflective of immunological changes in infected tissues. Examination of the mucosal responses, including local transcriptomic, microbiome and metabolic changes, immune infiltration, and changes in intestinal barrier function, should be made a priority of future research. Given the small number of participants in this study, it is possible that important immunological or microbial changes may have been missed. The small sample size also meant this study was not adequately powered to assess for differential effects of cryopreserved hookworm and fresh hookworm on the immune networks and microbiome. Findings from this study demonstrating the ability to infect participants with reanimated cryopreserved larvae will improve the feasibility of performing large clinical trials using controlled hookworm therapy.

In conclusion, this study has demonstrated that a viable infection of human hookworm can be established in all participants, including with reanimated cryopreserved hookworm larvae. These findings will assist in performing full-scale clinical trials using controlled hookworm therapy. Furthermore, this study has identified potential mechanisms by which hookworm may provide a beneficial effect on its host, including upregulation of CTLA-4 expression and tryptophan metabolism. While larger studies are needed to validate these findings, they provide valuable insight on the crosstalk between helminths and their host. Given the potential advantages for hookworm therapy as an alternative therapeutic option to conventional medication that may be favored by patients, further studies examining controlled hookworm therapy are warranted.

## Materials and methods

### Study design

This single-center, single-arm, study was performed to assess the viability and immunogenicity of hookworm (*Necator americanus*) larvae cultured from reanimated cryopreserved eggs and examine the immune cell, microbiome and metabolomic response to a controlled hookworm infection. It was conducted at the Malaghan Institute of Medical Research, Wellington, New Zealand, completed in accordance with the World Medical Association’s Declaration of Helsinki, approved by the Health and Disability Ethics Committee (Ethics reference 19/CEN/81) and registered with the Australian New Zealand Clinical Trials Registry (ACTRN12619001129178).

After informed consent, participants were screened for eligibility. Thirteen eligible participants received 30 hookworm larvae in the infective L3 lifecycle phase (L3), applied directly to the skin of the forearm. Nine individuals received hookworm larvae cultured from eggs freshly obtained from stool, while four individuals received hookworm larvae cultured from reanimated cryopreserved hookworm eggs. During the study, participants had scheduled study visits at baseline, and 2-, 4-, 6-, 8-, 12-, 16-, 24-, 36-, and 48-weeks post-infection, with unscheduled visits as required. At each of these visits the following were obtained: current symptoms including adverse events, physical examination, stool samples, and blood samples.

Participants discontinued the study if they developed recurrent mild adverse events (AE) or a serious adverse event (SAE) that in the investigators’ opinion would impact on the participants ability to continue the study, pregnancy, or request of the participant to withdraw. Those exiting the study completed a termination visit and were treated with anti-helminth therapy (mebendazole 100 mg BD for 3 days). A fecal egg count and blood eosinophils were checked 1 month following treatment to ensure successful eradication.

Participants remaining in the study at 48 weeks post-infection were given the opportunity to undertake a continuation phase where they were monitored every 4 to 12 weeks. Participants not undertaking the continuation phase were treated with mebendazole.

### Participant eligibility

Eligible individuals were 18–65 years old without a history of a significant respiratory, cardiovascular, gastroenterological, neurological, endocrine, or infectious disease. Participants were ineligible to participate if they had a current or previous helminth infection (other than *Enterobius vermicularis*); their stool contained enteric pathogens, Clostridium difficile toxin, or parasite ova; had been treated with antibiotics or anti-parasite medication in the last 6 months; had iron deficiency or anemia; had severe asthma that may require steroid use; had human immunodeficiency virus, hepatitis B or hepatitis C virus; had a history of cancer (excluding squamous cell carcinoma (SCC) or basal cell carcinoma (BCC) of the skin) within the last 5 years; current smokers; or had other clinically significant diseases that could interfere with the study protocol. Women needed a negative pregnancy test and be willing to practice birth control. Inclusion/exclusion criteria are displayed in Table S7.

### Hookworm preparation

Hookworm eggs were obtained from feces collected from volunteers chronically infected with *N. americanus* for this purpose. Two methods were employed to produce infective hookworm larvae. For ‘fresh’ larvae, eggs were extracted from freshly obtained feces, mixed with activated charcoal and incubated for 7–10 days at 26°C. For larvae originating from cryopreserved hookworm, cryopreserved L1 stage hookworm larvae were thawed and cultured into infective L3 stage larvae using techniques adapted from previously described methods with 0.08 M trehalose and 0.5 M dimethyl sulfoxide (DMSO) as the cryoprotectant.^24,25^ Larvae were isolated, washed in 5% iodine solution followed by sterile distilled water, and tested for morphological integrity and viability/motility by an experienced technician using dissecting microscopy. Each participant was infected with either 30 viable ‘fresh’ L3 larvae or 30 viable ‘cryopreserved’ L3 larvae by applying two dressings containing 15 larvae each to the forearms.

### Assessment of infection viability

The viability of the hookworm infection was assessed by quantifying hookworm eggs in stool from week 6 post-infection using light microscopy and direct hookworm visualization within the intestinal tract using the PillCamTM SB3 Capsule Endoscopy System (Medtronic). For the capsule endoscopy, participants were instructed to drink bowel prep (Klean-Prep), maintain a clear liquid diet from midday prior to administration and fast from midnight prior to administration. PillCamTM video recordings were analyzed using RAPID Reader software by two trained researchers.

### Assessment of symptoms

Possible symptoms attributable to a hookworm infection were assessed using a modified version of the Talley gut symptom questionnaire.^26^ Symptoms were graded as mild (nagging or annoying), moderate (strong negative influence on daily living), and severe (disabling) and were self-reported by each participant.

### Assessment of fecal markers of inflammation

For extraction ECP, EDN and HNL from feces the granule protein extraction protocol was used as described elsewhere.^27^ Feces samples were self-collected and immediately frozen at −80°C. On the day of processing feces samples were thawed overnight in the refrigerator or at RT for 1 h. Feces was weighted and diluted five times in extraction buffer consisting of phosphate-buffered saline, pH 7.4, supplemented with 10-mmol/L ethylenediaminetetraacetic acid, 0.2% N-cetyl- N,N,N-trimethylammonium bromide (CTAB) (Sigma), 20% glycerol (Sigma), 0.05% Tween20, and 1% bovine serum albumin. The mixture was homogenized using a homogenizer mixer until a homogenous solution was obtained. After incubation at 4°C for 30–45 min and mixing, the homogenate was centrifuged at 4,000 g for 30 min at 5°C. The supernatant was aliquoted and frozen at −20°C for later ELISA analysis. For each sample, a separate tube of the diluted homogenate was weighed and centrifuged. By weighing the pellet obtained after discarding the supernatant, a measure of semidry weight was obtained. Faecal EDN and HNL were measured by ELISA (Diagnostics Development AB, Uppsala, Sweden) according to the manufacturer’s protocol. The levels of markers in feces were adjusted for water content and expressed as micrograms per gram of semidry feces. For extraction of fecal calprotectin CALEX Cap devices (Bühlmann Laboratories, USA) were used according to the manufacturer’s protocol. Fecal calprotectin was measured by fCal ELISA (Bühlmann Laboratories, USA) according to the manufacturer’s protocol.

### Whole blood analysis

Whole blood immunophenotyping was performed using 600 µL of heparinized whole blood. RBCs were lysed with ACK Lysis Solution (ddH_2_O with 1 mm EDTA, 150 mm NH_4_Cl, 10 mm NaHCO_3_ at pH 7.4) at 4°C for 10 min. Samples were diluted in PBS to arrest cell lysis and centrifugation at 500 g for 5 min at 4°C. Cells were then washed with PBS, counted, and stained with 1:2000 Zombie NIR Fixable Viability dye (Biolegend) at room temperature for 15 min and protected from light. Cells were then stained with the antibody mix (Data file S2) in FACS buffer (PBS +2% FBS, 0.01% NaN₃) and 20% Brilliant Stain Buffer Plus (BD Biosciences), at 4°C for 20 min. Finally, cells were washed twice with FACS buffer and analyzed on a 5-laser Aurora spectral flow cytometer (Cytek Biosciences). Data was analyzed using FlowJo v10.7.1 and Prism V8 (GraphPad Software Inc.).

### Cytokine and chemokine analysis

Plasma was separated from heparinized whole blood by centrifugation at 2000 g for 15 min at 4°C. Serum was separated from clotted blood by centrifugation at 2000 g for 10 min at 4°C. Plasma and serum were stored at −80°C until analysis. Using the Luminex Multiplex Assay, serum samples were tested for IgE, IgG isotypes, IgA, IgM, RANTES, BCA-1, Eotaxin-3, TSLP, IL-33, eotaxin, IFNγ, IL-1β, IL-3, IL-4, IL-5, IL-6, IL-8, IL-10, IL-12.p70, IL-13, IL-17A, TNFα, and TGF-β, according to the manufacturer’s instructions. Cytokines under the limit of detection are not shown in the result section. For *Na*-specific IgG ELISA, samples were analyzed following the protocol previously described.^28^

### PBMC isolations and analysis

Peripheral blood mononuclear cells (PBMCs) were isolated from heparinized whole blood using SepMate™ PBMC Isolation Tubes (STEMCELL Technologies). Collected PBMCs were washed three times with PBS + 2% FBS and cryopreserved in FBS + 10% DMSO. Cryovials containing PBMC suspension were transferred to a Corning^Ⓡ^ CoolCell ^Ⓡ^ Containers and placed at −80°C overnight. Cryovials were then stored in liquid nitrogen until analysis. On day of analysis, thawed cells were resuspended in PBS + 2% FBS, counted, and up to 5 × 10^6^ cells were resuspended in FACS buffer for staining. Two antibody panels were designed to assess 1) T cell subsets, NK T cells, MAIT cells and 2) B cells, NK cells, Monocytes and Dendritic cells. Details on antibodies used and gating strategies are listed in Table S8 and Figure S4. PBMCs were incubated with 1:2000 Zombie NIR Fixable Viability dye (Biolegend) at room temperature for 20 min. After washing, cells were incubated with 1:40 FC block (Biolegend) for 10 min at room temperature.

For panel 1, cells were surface stained for CD1d and MR1 tetramers for 15 min at 37°C, washed and sequentially stained for CD25, TCRgd, CXCR3, CCR10, CCR6, CCR4, CCR7 for 5 min each at 37°C. For panel 2, cells were sequentially stained for CXCR5 and CCR7 for 5 min each at 37°C. After sequential staining, cells were incubated with surface receptor mix for 20 min at room temperature in the presence of Brilliant Buffer Plus (BD Biosciences) and Monocyte Block (1:30, Biolegend) only for panel 2. Cells were fixed and permeabilized with FoxP3/Transcription Factor Staining Buffer Set (eBioscience) for 30 min at room temperature and subsequently incubated with FC block (1:40, Biolegend) for 10 min at room temperature. Cells were washed in Permeabilization Buffer (eBioscience), incubated with intracellular antibody mix for 40 min on a shaking plate at room temperature. Cells were then resuspended in FACS buffer and analyzed on a 5-laser Aurora spectral flow cytometer (Cytek Biosciences).

### Microbiome analysis

Fecal samples were collected using a disposable toilet accessory (DNA Genotek, Canada) and placed into OMNIgene. GUT tubes (DNA Genotek, Canada) according to the manufacturer’s instructions. Samples were immediately cooled, delivered to the laboratory within 24 h of collection, and stored at −80°C until processed. DNA was extracted from fecal samples using the Qiagen MagAttract PowerSoil DNA KF Kit optimized for the Thermofisher KingFisher robot. DNA quantification and quality checks were done via Qubit. 16s rDNA marker gene (V4) was amplified using the high-fidelity Phusion polymerase using dual-barcoded primers^29^ and PCR products were verified by gel electrophoresis. PCR reactions were cleaned and normalized using the SequalPrep 96-well Plate Kit. Library samples were pooled and sequenced on the Illumina Miseq. Fastq files were quality filtered and clustered into 97% similarity operational taxonomic units (OTUs) using the mothur software package.^29^ Representative OTU sequences were assigned taxonomic classification using a Bayesian classifier against the Ribosomal database project (RDP) database.^30^ Before filtering, alpha diversity (Shannon measure) was calculated and plotted using Phyloseq.^31^ Data were centered log-ratio (CLR) transformed and filtered. Data was further analyzed in R using custom scripts and plotted using ggplot2.^32^

### Plasma metabolomics analysis

Metabolomics was performed on plasma samples from pre-infection, the acute infection phase (week 8 or 12 post-infection) and the chronic phase (week 36 post-infection). Polar metabolites were extracted from participant plasma samples as previously described.^33^ Briefly, processed plasma was placed into an HPLC vial for LC-MS using HILIC chromatography. Pooled quality control (QC) samples were prepared by pooling together 50 µL of each participant plasma sample. QC were analyzed alongside samples to allow for signal normalization to correct for any loss of signal across the analytical batch. Polar metabolites extracted from plasma samples were analyzed using a Shimadzu LCMS-9030 mass spectrometer equipped with a Shimadzu Nexera-x2 UHPLC system. Data were captured, converted, and peaks were then annotated using the DIA MS/MS data collected against the built-in human metabolite library containing 13,303 MS/MS spectra.^34^ The resultant peak intensity table underwent run-order correction and normalization using pooled QC samples and the locally weighted scatterplot smoothing (LOWESS) regression model. Plasma metabolome data (Data File S1) underwent log-transformation and autoscaling prior to the following analyses: (i) Metabolite Set Enrichment Analysis (MSEA) using the Quantitative Enrichment Analysis (QEA) module on MetaboAnalyst 5.0^35^; (ii) principal component analysis (PCA); (iii) permutational multivariate analysis of variance (PERMANOVA); (iv) mixed-mode repeated measures two-way ANOVA with Dunnett’s post-hoc multiple comparison tests, all analyzed using R software and/or GraphPad Prism Version 9. Additional metabolomics methodology in supporting information.

### Data analysis

Whole blood flow cytometry data were analyzed using FlowJo software. PBMCs flow cytometry data were normalized using CytoNorm algorithm in R studio and analyzed using flowSOM clustering algorithm and UMAP algorithm as a visualization tool using the cloud-based OMIQ software (San Francisco, USA). Heatmaps and PCA plots were generated using R software or OMIQ and modified in Adobe Illustrator. *p* values less than 0.05 were considered statistically significant. All statistical analyses were performed using GraphPad Prism 9 package (GraphPad Software, Inc., San Diego, CA) or R 4.1 (R Institute, Vienna).

## Supplementary Material

Supplemental Material

## Data Availability

Data openly available in a public repository Microbiome Data: https://zenodo.org/records/7742942. (fastq files) and https://zenodo.org/records/7743294. (metadata) Metabolomics data: are available https://zenodo.org/records/8339501.
