# Supplementary material for: Controlled infection with cryopreserved human hookworm induces CTLA-4 expression on Tregs and upregulates tryptophan metabolism
Source: Gut Microbes. 2024 Oct 16;16(1):2416517. doi: 10.1080/19490976.2024.2416517 (PMC11485773; doi:10.1080/19490976.2024.2416517)
Supplement: Supplemental Material [file KGMI_A_2416517_SM1620.zip › Supporting information - 2416517/kgmi-s-2024-1028-20241010194408/suppl_data/Supplementary.docx]

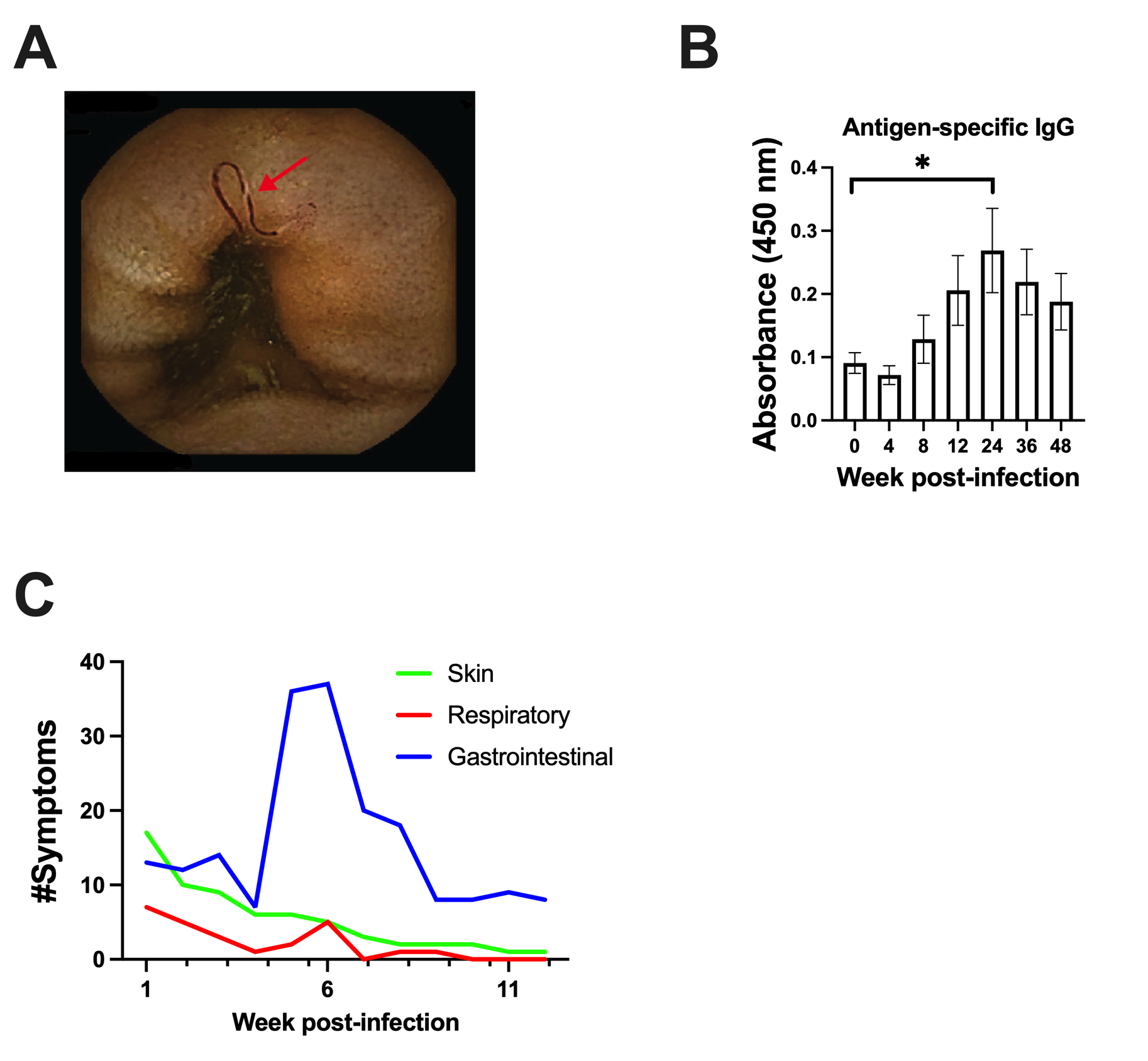


**Figure S1 Skin images and changes in serum and stool cytokines**

(A) Representative images of Pillcam^TM^ endoscopy showing hookworm (red arrow) in the intestinal tract. (B) Bar graph showing mean ± SEM of antigen-specific IgG, measured by antigen-specific ELISA. Analyzed using One-way ANOVA with mixed-effects analysis with Tukey’s multiple comparison test. p= *<0.05 (C) Number of total symptoms reported by all the participants divided by organs affected by parasite traverso in the host.


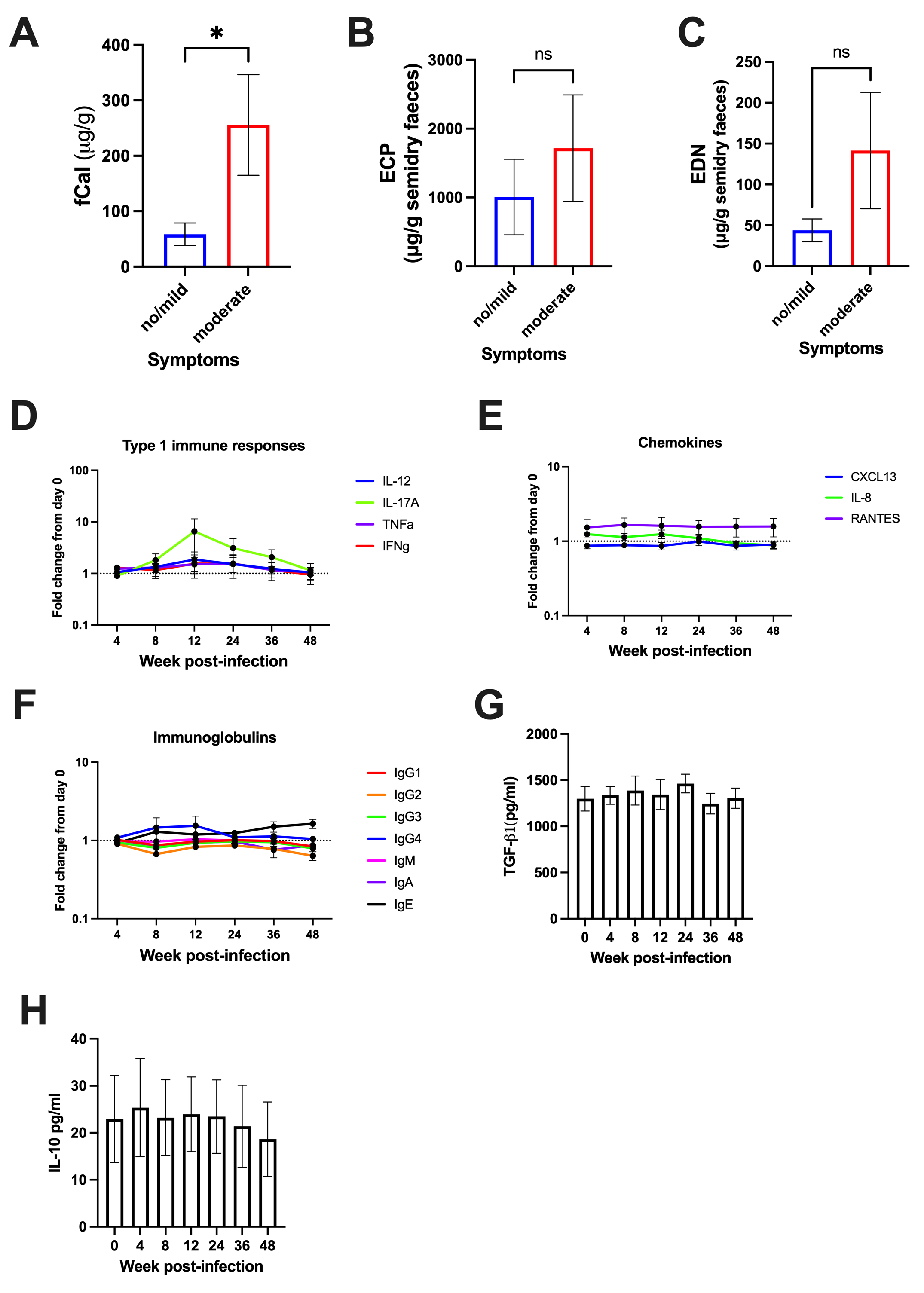


**Figure S2.** (A-C) Mean ± SEM of eosinophils count (G), fCal (H), ECP (I) and EDN (J) according to symptoms severity. (D-F) Graph showing fold changes from baseline (day 0 – uninfected) of type 1 cytokines (B), chemokines (C) and immunoglobulins (D) over the course of infection. (G-H) Bar graph showing mean ± SEM of TGF-*β*1 and IL-10 measured by Luminex (N=8). Analyzed using One-way ANOVA with mixed-effects analysis with Tukey’s multiple comparison test. p= *<0.05


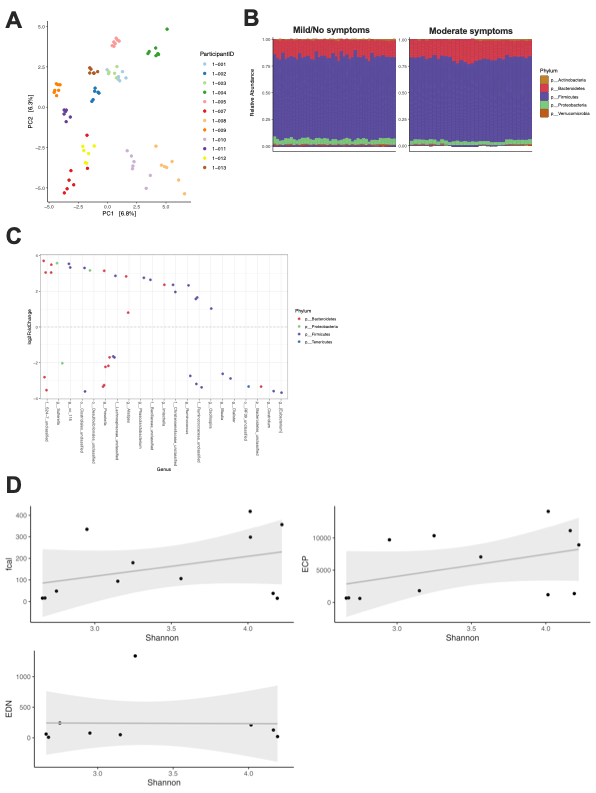


**Figure S3. Microbial changes during hookworm infection**

(A) PCA plots showing individual participants microbiome per time point. (B) Phylum relative abundance for participants with mild/no symptoms (0) and moderate symptoms (1). (C) OTUs that were identified to be significantly different between those who experience mild to moderate symptoms (symptom score 0) and those who experience severe symptoms (symptom score 1) as determined using DESeq2 with an adjusted P value < 0.01. (D) Spearman correlation between an individual’s baseline microbiome diversity (Shannon index) and fCal, faecal ECP and faecal EDN levels.


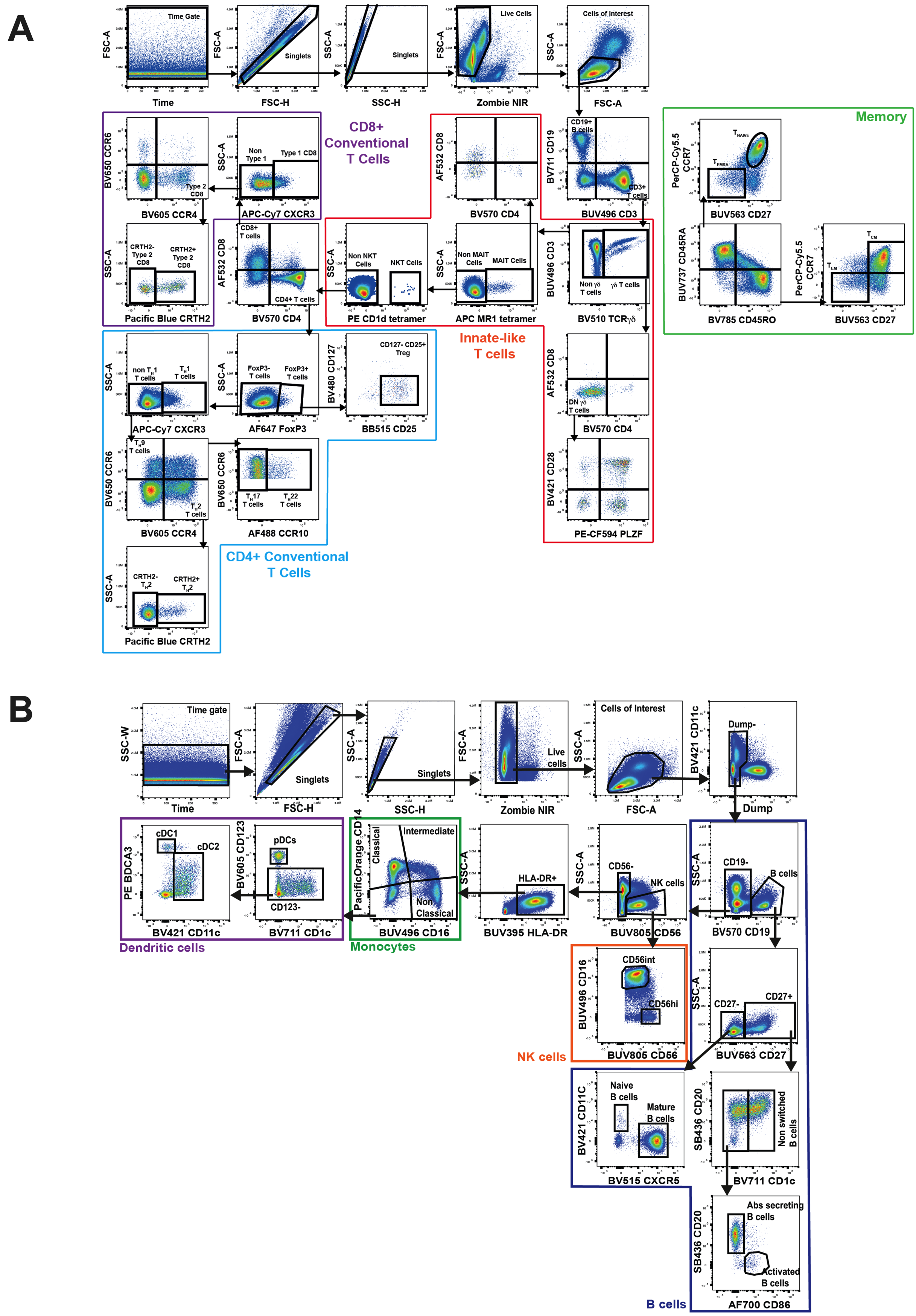


**Figure S4. Expert gating used to identify PBMC populations**

Representative flow plots of the expert gating strategies used during panel 1(A) and panel 2(B) development and assessment.

**Figure S5. Changes in immune cells during hookworm infection**

(A) Representative UMAP marker expression for panel 1. (B) Bar graph showing mean ± SEM for each immune cell populations identified using panel 1. (C) Heatmap showing percentage of cluster identified using FlowSOM for panel 1. (D) Representative flow plots gated on CD4+ T cells showing CCR7 vs CD45RA subsets. CCR7+ CD45RA+ cells were gated and showing CD127+ cells. (E) Graph showing percentage of CCR7+ CD45RA+ T cells. (F) Graph showing CD127 MFI for the CCR7+ CD45RA+ population. (G) Percentage of CD127+ cells of CCR7+ CD45RA+ T cells. For (E-F-G) the three coloured line highlights the three patients with increased CCR7+ CD45RA+ T cells. (H) Representative UMAP marker expression for panel 2. (I) Bar graph showing mean ± SEM for each immune cell populations identified using panel 2. (K) Heatmap showing percentage of cluster identified using FlowSOM for panel 2. (J) CTLA-4 MFI measured for CD4+ CCR4+ CXCR3- Th2 cells. (L) CTLA-4 MFI measured for CCR6+ CCR4- CXCR3- Th9 cells. (M-N) Bar graph showing percentage of CTLA-4+ cells for the population in J and L respectively. Results were analysed with One-way ANOVA with Mixed-effect analysis with Tukey’s multiple comparison test. p= *<0.05, **<0.005, ***< 0.0005.


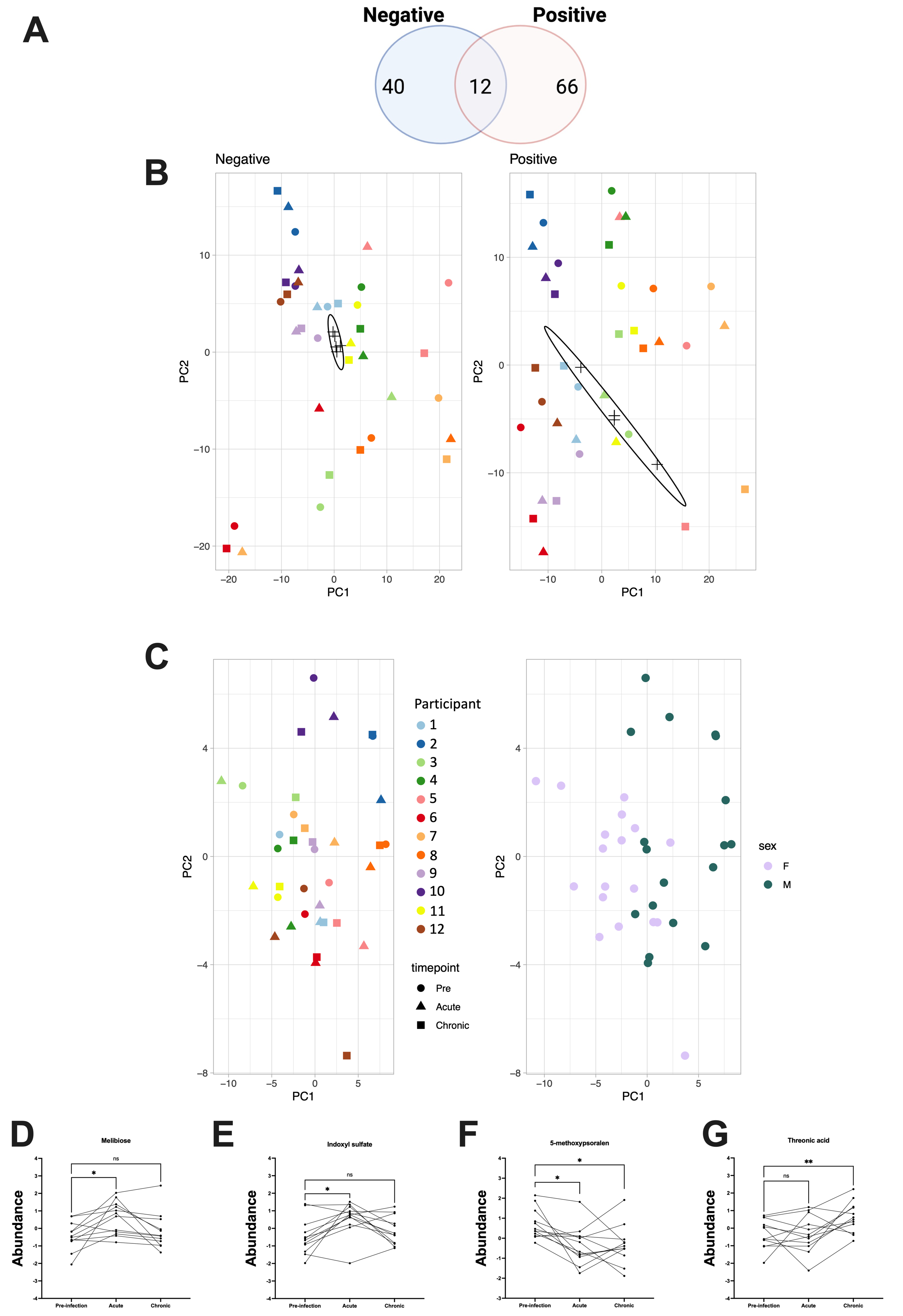


**Figure S6. Metabolic changes during hookworm infection**

A) Number of annotated plasma metabolites detected in the negative or positive electrospray ionization (ESI) modes, including those detected in both modes. (B) Data overview and quality assurance demonstrating good method reproducibility in the negative (left panel) and positive ESI (right panel) modes, as reflected by tight clustering of QCs evident in the principal component analysis (PCA) scores plots. (C) PCA scores plot on the plasma polar metabolome labelled by participant ID, by sex.

**Table S1:** Microbiome Dataset Phylum and Symptoms score

| Phylum | statistic | df | p | p.adj | p.adj.signif |
| --- | --- | --- | --- | --- | --- |
| p__Bacteroidetes | -6.2301264 | 65.6146685 | 3.76E-08 | 4.89E-07 | **** |
| p__Proteobacteria | 5.54182625 | 69.5253937 | 5.01E-07 | 3.26E-06 | **** |
| p__Tenericutes | 3.73818076 | 62.2403532 | 0.000405 | 0.001755 | ** |
| p__Crenarchaeota | 2.70101991 | 70.8696479 | 0.00864 | 0.02808 | * |
| p__Chloroflexi | 2.54728959 | 73.9434556 | 0.0129 | 0.03354 | * |
| p__Acidobacteria | 1.44430625 | 72.3362745 | 0.153 | 0.28971429 | ns |
| p__Verrucomicrobia | 1.43443764 | 73.159074 | 0.156 | 0.28971429 | ns |
| p__Cyanobacteria | 1.24151836 | 73.9401441 | 0.218 | 0.35425 | ns |
| p__Firmicutes | -1.1602273 | 69.6448072 | 0.25 | 0.36111111 | ns |
| p__Lentisphaerae | 0.48327729 | 73.7755738 | 0.63 | 0.819 | ns |
| p__Actinobacteria | 0.33662103 | 69.4034727 | 0.737 | 0.871 | ns |
| p__Euryarchaeota | 0.15240284 | 73.9762936 | 0.879 | 0.912 | ns |
| p__Gemmatimonadetes | -0.1106562 | 67.8912465 | 0.912 | 0.912 | ns |

**Table S2:** Differentially abundant taxa in participants with moderate symptoms

**Table S3. Metabolic Set Enrichment Analysis (MSEA) statistics comparing metabolic pathways enriched during the acute infection phase, relative to baseline (pre-infection phase)**

| **No** | **Metabolic pathway** | **Total compounds^a^** | **Hits** | **Q-statistic** | **Expected  Q-Statistic** | **Enrichment Ratio^b^** | **Nominal P-value** | **Holm P-value** | **FDR^c^** |
| --- | --- | --- | --- | --- | --- | --- | --- | --- | --- |
| 1 | Galactose metabolism | 27 | 1 | 27.77 | 4.35 | 6.388 | 0.0081 | 0.285 | 0.285 |
| 2 | Tryptophan metabolism | 41 | 3 | 12.26 | 4.35 | 2.821 | 0.0496 | 1 | 0.581 |
| 3 | D-glutamine & D-glutamate metabolism | 6 | 1 | 13.04 | 4.35 | 2.999 | 0.083 | 1 | 0.581 |
| 4 | Glyoxylate & dicarboxylate metabolism | 32 | 1 | 13.04 | 4.35 | 2.999 | 0.083 | 1 | 0.581 |
| 5 | Nitrogen metabolism | 6 | 3 | 13.04 | 4.35 | 2.999 | 0.083 | 1 | 0.581 |
| 6 | Alanine, aspartate and glutamate metabolism | 28 | 2 | 6.84 | 4.35 | 1.574 | 0.196 | 1 | 0.809 |

**^a^** Total number of metabolites deposited in the KEGG metabolic pathway database
**^b^ Enrichment ratio:** Ratiometric enrichment expressed as acute phase/pre-infection phase
**^c^** P-value adjusted for False discovery rate (FDR) according to the procedure of Benjamin and Hochberg *(57)*

**Table S4. Metabolic Set Enrichment Analysis (MSEA) statistics comparing metabolic pathways enriched during the chronic infection phase, relative to baseline (pre-infection phase)**

| **No** | **Metabolic pathway** | **Total compounds^a^** | **Hits** | **Q-statistic** | **Expected  Q-Statistic** | **Enrichment Ratio** | **Nominal P-value** | **Holm P-value** | **FDR^b^** |
| --- | --- | --- | --- | --- | --- | --- | --- | --- | --- |
| 1 | Phenylalanine, tyrosine and tryptophan metabolism | 4 | 2 | 8.63 | 4.35 | 1.985 | 0.149 | 1.00 | 0.612 |
| 2 | Selenocompound metabolism | 20 | 1 | 9.08 | 4.35 | 2.089 | 0.152 | 1.00 | 0.612 |
| 3 | Ubiquinone and other terpenoid-quinone biosynthesis | 9 | 1 | 7.66 | 4.35 | 1.762 | 0.190 | 1.00 | 0.612 |
| 4 | Tyrosine metabolism | 42 | 1 | 7.66 | 4.35 | 1.762 | 0.213 | 1.00 | 0.612 |
| 5 | Phenylalanine metabolism | 10 | 3 | 6.54 | 7.66 | 1.387 | 0.246 | 1.00 | 0.612 |
| 6 | Tryptophan metabolism | 41 | 3 | 6.03 | 7.66 | 1.339 | 0.256 | 1.00 | 0.612 |

^a^ Total number of metabolites deposited in the KEGG metabolic pathway database
^b^ Enrichment ratio: Ratiometric enrichment expressed as chronic phase/pre-infection phase
^c^ P-value adjusted for False discovery rate (FDR) according to the procedure of Benjamin and Hochberg

**Table S5. PERMANOVA Analysis identifying “Sex” and “Participant ID” as important covariates to control for in downstream statistical analyses**

| **Factor** | **Df** | **Sum of Squares** | **R^2^** | **F** | **Pr(>F)** |
| --- | --- | --- | --- | --- | --- |
| Sex | 1 | 0.12153 | 0.18997 | 7.9168 | 0.001*** |
| Participant ID | 1 | 0.02956 | 0.04621 | 1.9257 | 0.072 |
| Timepoint | 1 | 0.00865 | 0.01351 | 0.5632 | 0.792 |
| participantID:timepoint | 1 | 0.00412 | 0.00645 | 0.2686 | 0.986 |
| Residual | 31 | 0.47589 | 0.74386 |  |  |
| Total | 35 | 0.363975 | 1 |  |  |

**Significant codes:** p < 0***, p < 0.001**, p < 0.05*

**Table S6:** **Two-way, repeated-measures Mixed ANOVA analysis, controlling for “sex” and “participant ID” as covariates.**

| **No** | **Variables** | **Effect** | **DFn** | **DFd** | **F** | **p** | **p<.05** | **ges** | **p.adj** |
| --- | --- | --- | --- | --- | --- | --- | --- | --- | --- |
| **1** | Kynurenine | timepoint | 2 | 33 | 5.108 | 0.012 | * | 0.236 | 0.5076 |
| **2** | Melibiose | timepoint | 2 | 33 | 4.046 | 0.027 | * | 0.197 | 0.5076 |
| **3** | Threonic.acid | timepoint | 2 | 33 | 4.089 | 0.026 | * | 0.199 | 0.5076 |
| **4** | Indoxyl sulfate | timepoint | 2 | 33 | 4.1 | 0.026 | * | 0.199 | 0.5076 |
| **5** | Methoxypsoralen | timepoint | 2 | 33 | 5.503 | 0.009 | * | 0.25 | 0.5076 |

**Table S7: Participant inclusion/exclusion criteria**

| **Participant Inclusion Criteria** |
| --- |
| Participant has provided written informed consent and is willing to comply with all Protocol scheduled visits, laboratory tests, and other trial procedures and in the opinion of the Investigator has a good understanding of the Protocol, the length of the study and the demands of the study. |
| Participants will be male and non-pregnant, non-lactating females aged between 18 to 65 years. |
| Participants must weigh more than 50kg with a BMI within the 18 – 35kg/m^2^ range |
| Participants must understand the procedures involved and agree to participate in the study by giving fully informed, written consent prior to any study assessment. |
| Participants must be contactable and available for the duration of the clinical trial. |
| If female, has met either of criterion “a or “b” below:  **(a) If of non-childbearing potential**, has met 1 of the following – Amenorrhoeic for at least 2 years, or has had a hysterectomy and/or bilateral oophorectomy at least 8 weeks prior to screening, or has had a tubal ligation at least 8 weeks prior to screening.  **(b)** **If of childbearing potential**, must be willing to use the acceptable methods of contraception |
| In the opinion of the investigator is in good general health |
|  |
| **Participant Exclusion Criteria** |
| Current or history of helminth infection (other than *E. vermicularis*). |
| Have any finding at screening that in the opinion of the Investigator or medical monitor would compromise the safety of the Participant or affect their ability to adhere to protocol scheduled visits, treatment plan, laboratory tests, and other trial procedures. |
| Have participated in any other clinical trial and/or have received an investigational drug or device within 30 days of screening. |
| History or current evidence of any of the following: compromised respiratory function (chronic obstructive pulmonary disease, respiratory depression, signs or symptoms of hypoxia at screening); thyroid pathology (unless stabilized and euthyroid for >3 months at the time of screening); hepatitis B, hepatitis C, or human immunodeficiency virus (HIV) infection; evidence of clinically significant chronic cardiac, hepatic or renal disease; psychiatric illness (poorly controlled); seizure disorder or any other chronic health issues that in the opinion of the Investigator would exclude the Participant from the trial. |
| Have one of the following laboratory abnormalities: ferritin <20 μg/L, transferrin <2.04 g/L or Hb <120 g/L for females or 130 g/L for males.  History of severe asthma or other health conditions that may require future steroid use; |
| History of substance abuse or current substance abuse that in the opinion of the Investigator would exclude the Participant from the trial. |
| History of intolerance, allergy or hypersensitivity to the proposed anthelmintic – mebendazole. |
| History of intolerance, allergy or hypersensitivity to the Betadine (iodine) solution used in preparation of *N. americanus* that in the opinion of the Investigator would exclude the participant from the trial. |
| History of malignancy of any organ system (other than localized basal cell carcinoma of the skin), treated or untreated, within the past 5 years. |
| For female subjects: positive urine pregnancy test at screening. |
| Current or past scars, tattoos, or other disruptions of skin integrity at the intended site of larval application. |
| Poor venous access making the Participant unable to comply with the safety laboratory testing requirements. |
| Probiotic or prebiotic supplementation within 1 month of screening or commencement during the study |
| Significant dietary change or weight loss (>5%) within 6 months of screening or during the study |
| Smokers or high alcohol consumers |

**Table S8:List of flow cytometry antibodies**

| **Whole Blood Antibodies** |  |  |  |  |
| --- | --- | --- | --- | --- |
| **Antigen** | **Fluorophore** | **Clone** | **#Cat** | **Supplier** |
| CD19 | BUV805 | HIB19 | 742007 | BD |
| FceRIa | BV421 | AER-37 (CRA-1) | 334624 | Biolegend |
| CD10 | BV480 | HI10a | 746847 | BD |
| CD56 | BV570 | HCD56 | 318330 | Biolegend |
| CD123 | BV605 | 6H6 | 306026 | Biolegend |
| CD11b | BV650 | ICRF44 | 301336 | Biolegend |
| CD15s | BV711 | CSLEX1 | 563910 | BD |
| CD4 | BV750 | SK3 | 344644 | Biolegend |
| Siglec8 | BV786 | 837535 | 747868 | BD |
| HLADR | FITC | G46-6 | 307604 | Biolegend |
| CD3 | AF532 | UCHT1 | 58-0038-42 | Thermofisher |
| CD16 | PerCP | 3G8 | 302030 | Biolegend |
| CXCR4 (CD184) | PE-CF594 | 12G5 | 562389 | BD |
| CD8 | PE-Cy5 | RPA-T8 | 301010 | Biolegend |
| CD203c | APC | NP4D6 | 324610 | Biolegend |
| CD14 | AF700 | HCD14 | 325614 | Biolegend |
| CD15 | APC-Cy7 | W6D3 | 323048 | Biolegend |
|  |  |  |  |  |
| **PBMC Antibodies** |  |  |  |  |
| **Antigen** | **Fluorophore** | **Clone** | **#Cat** | **Supplier** |
| CCR10 | AF488 |  | RDSFAB3478G0100 | R&D Systems |
| CD11c | BV421 | 3.9 | 301628 | Biolegend |
| CD123 | BV605 | 6H6 | 306026 | Biolegend |
| CD127 | BV480 | HIL-7R-M21 | 566101 | BD Biosciences |
| CD14 | PacOrange | TuK4 | MHCD1430 | ThermoFisher |
| CD141 (BDCA3) | PE | AD5-14H12 | 130-113-318 | Miltenyi Biotec |
| CD15 | PE-Cy7 | W6D3 | 323030 | Biolegend |
| CD16 | BUV496 | 3G8 | 612944 | BD Biosciences |
| CD183 (CXCR3) | APC-Cy7 | G025H7 | 353722 | Biolegend |
| CD185 (CXCR5) | BB515 | RF8B2 | 564624 | BD Biosciences |
| CD19 | BV570 | HIB19 | 302236 | Biolegend |
| CD19 | BV711 | Sj25C1 | \| 563036 \| \| --- \| | BD Biosciences |
| CD194 (CCR4) | BV605 | L291H4 | 359418 | Biolegend |
| CD196 (CCR6) | BV650 | 11A9 | 563922 | BD Biosciences |
| CD197 (CCR7) | PerCP-Cy5.5 | G043H7 | 353220 | Biolegend |
| CD1c | BV711 | L161 | 331536 | Biolegend |
| \| CD20 \| \| --- \| | SB436 | 2H7 | 62-0209-42 | ThermoFisher |
| CD203c | PE-Cy7 | NP4D6 | \| 324618 \| \| --- \| | Biolegend |
| CD25 | APC-Fire750 | BC96 | 302642 | Biolegend |
| CD25 | BB515 | 2A3 | \| 564467 \| \| --- \| | BD Biosciences |
| CD27 | BUV563 | M-T271 | 741336 | BD Biosciences |
| CD279 (PD-1) | BV480 | EH12.1 | 566112 | BD Biosciences |
| \| CD28 \| \| --- \| | BV421 | CD28.2 | 302930 | Biolegend |
| CD294 (CRTH2) | PacBlue | BM16 | \| 350130 \| \| --- \| | Biolegend |
| CD3 | BUV496 | UCHT1 | 612940 | BD Biosciences |
| CD3 | PE-Cy7 | UCHT1 | 300420 | Biolegend |
| CD4 | AF532 | RPA-T4 | \| 58-0049-42 \| \| --- \| | ThermoFisher |
| CD4 | BV570 | RPA-T4 | 300534 | Biolegend |
| CD45RA | BUV737 | HI100 | 612846 | BD Biosciences |
| CD45RA | BV650 | HI100 | 304136 | Biolegend |
| CD45RO | BV785 | UCHL1 | 304234 | Biolegend |
| CD56 | BUV805 | NCAM16.2 | \| 749086 \| \| --- \| | BD Biosciences |
| CD8 | AF532 | RPA-T8 | 58-0088-42 | ThermoFisher |
| CD80 | BV510 | 2D10 | 305234 | Biolegend |
| CD83 | BUV737 | HB15E | \| 612823 \| \| --- \| | BD Biosciences |
| CD86 | AF700 | FUN-1 | 561124 | BD Biosciences |
| CTLA-4 | PE-Cy7 | 14D3 | 25-1529-42 | ThermoFisher |
| CTLA-4 | PerCP-eFluor710 | 14D3 | 46-1529-42 | ThermoFisher |
| FceR1a | AF647 | AER-37 | 334614 | Biolegend |
| FoxP3 | AF647 | 259D | 320214 | Biolegend |
| HLA-DR | BUV395 | G46-6 | 564040 | BD Biosciences |
| PLZF | PE-CF594 | R17-809 | \| 565738 \| \| --- \| | BD Biosciences |
| TCRgd | BV510 | B1 | 331220 | Biolegend |
| CD1d Tetramer | PE | BS-57 | 53235 | NIH Tetramer Core Facility |
| MRI Tetramer | APC | 5-OP-RU | 53233 | NIH Tetramer Core Facility |
| Zombie NIR Viability Dye |  |  |  | Biolegend |
|  |  |  |  |  |

**Supplementary Methods – Metabolomics**

**Liquid Chromatography and mass spectrometry instrumental conditions**

All solvents and reagents used for extracting polar metabolites from plasma; including mobile phases used for untargeted liquid chromatography mass spectrometry (LC-MS) analyses (CH_3_CN, HCOOH) were purchased from ThermoFisher Scientific (Auckland, New Zealand). MilliQ® type I ultrapure water was purchased from Merck Millipore (Bedford, MA, USA). HPLC-grade Ammonium formate (Fluka™) was purchased from Sigma-Aldrich (Auckland, New Zealand). The Thermo Accucore HILIC column (2.1 x 100 mm, 2.6 μm particle size) was purchased from Thermo Fisher Scientific. Polar metabolites were extracted from participant plasma samples as previously described^1^. Briefly, for each plasma sample, 50 μL was diluted tenfold with pre-chilled acetonitrile/water (9:1 v/v), vortexed (1 min, 30 Hz) and then centrifuged for 10 mins (13,663 *x* g, 4°C). 200 µL of supernatant was placed into an HPLC vial for LC-MS using HILIC chromatography, as detailed below. Solvent blanks were also extracted using the same method and analysed to monitor any potential sample carryover issues during LC-MS analyses. Pooled quality control (QC) samples were prepared by pooling together 50 µL of each participant plasma sample. QC samples underwent extraction by the same method described above and analyzed alongside samples to allow for signal normalization to correct for any loss of signal across the analytical batch.

Polar metabolites extracted from plasma samples were analysed using a Shimadzu LCMS-9030 mass spectrometer equipped with a Shimadzu Nexera-x2 UHPLC system. Metabolites were measured by injecting 5 μL of plasma supernatant onto the Thermo Accucore HILIC column (2.1 x 100 mm, 2.6 μm particle size). Samples were held in the autosampler at 4 °C and the column oven held at 30 °C. Metabolites were eluted over a 23-min linear gradient with a flow rate of 400 μL/min. The mobile phases were water containing 10 mM ammonium formate: 0.1% formic acid (solvent A) and acetonitrile containing 0.1% formic acid (solvent B). The gradient elution programme was as follows: 90% B (0–1 min), 90–70% B (1.0–11.5 min), 70–10% B (11.5–13.5 min) and held at 10% B for 0.5 min (13.5–14.0 min) before returning to 90% B (14.0–14.1 min) and held to re-equilibrate until 16 min. The first 0.5 min and the last 1 min of the chromatographic run were diverted to waste to keep the MS ion source clean. The mass spectrometer was operated in both positive and negative ionisation modes (separate analyses), measuring full MS1 spectra from 55-1100 m/z across the entire chromatogram and also collecting Data Independent Acquisition (DIA) data in 20 m/z windows from 90-910 m/z, with a 0.6 sec cycle time and collision energy of 23 normalised collision energy units. The source voltage was +4.0kV with a nebulising gas flow of 2.0 L/min, heater gas flow of 10 L/min, interface temperature of 300 °C, drying gas flow of 10 L/min, desolvation line temperature of 250°C and a heater block temperature of 400 °C. All drying and collision gasses used were nitrogen.

A total of 580 and 541 raw MS features were respectively detected in both the negative and positive electrospray ionization (ESI) modes, measured across all participant samples. Of these detected features, 40 and 66 MS features in the ESI negative and ESI positive modes, respectively, were annotated based on mass spectral database matching with MS-DIAL’s built-in metabolomics databases. Twelve annotated metabolites including amino acids (arginine, asparagine, cystine, glutamine, histidine phenylalanine, serine, threonine, tyrosine), amino acid metabolic byproducts (citrulline, phenylacetylglutamine) and haem metabolism (bilirubin) were detected in both ESI modes. In downstream functional analyses, only the data in the best-performing ESI mode were included for each of the 12 metabolites. Thus, a total of 94 unique metabolites were annotated, broadly encompassing a diversity of chemical classes including: amino acids, nucelotides, amino acid metabolism products, organic acids, some dietary-related alkaloids and exogenous drugs (e.g. paracetamol). Investigation of the sample variation after correction for run-order using QC samples highlighted excellent quality of the data across both positive and negative ESI modes, as evidenced by tight clustering of pooled participant QC samples in the central area of the plot and well within the variance of the samples, collectively demonstrating no major run order effects in the data

1. Fraser K, Roy NC, Goumidi L, et al. Plasma Biomarkers and Identification of Resilient Metabolic Disruptions in Patients With Venous Thromboembolism Using a Metabolic Systems Approach. Arterioscler Thromb Vasc Biol. 2020 Oct;40(10):2527-2538.
